# Re-orienting transdisciplinary research and community-based participatory research for health equity

**DOI:** 10.1017/cts.2022.15

**Published:** 2022-02-04

**Authors:** Sarah D. Hohl, Marian L. Neuhouser, Beti Thompson

**Affiliations:** 1 School of Public Health, University of Washington, Seattle, WA, USA; 2 Public Health Sciences Division, Fred Hutchinson Cancer Research Center, Seattle, WA, USA; 3 Department of Family Medicine and Community Health, Office of Community Health, University of Wisconsin-Madison, Madison, WI, USA

**Keywords:** Health equity, health disparities, team science, community engagement, transdisciplinary collaboration

## Abstract

**Introduction:**

Transdisciplinary (TD) research and community-based participatory research (CBPR) represent promising investigative approaches to ameliorate health disparities. Public investments in team-based TD research to address multifactorial public health problems have increased over the last two decades. Similarly, recognition that community participation in research and social action is essential to promoting health equity is reflected in increased prioritization of community engagement in research and practice. Yet, models that describe and guide the combined TD and CBPR approach are lacking.

**Methods:**

We utilized a qualitative, convergent parallel case study design that included document reviews and one-on-one interviews to assess how investigators from the Centers for Population Health and Health Disparities (CPHHD) initiative integrated TD team science and CBPR in their work, and what they perceived as the impact of that work on health equity.

**Results:**

Twenty-five CPHHD investigators and National Institutes of Health program staff participated in a one-on-one interview. Document and interview data informed the development of an iterative conceptual model of TD CBPR comprising five domains: problem focus, contexts, collaboration and partnership, outcomes, and societal impact of TD CBPR.

**Conclusions:**

TD team science and CBPR are integrally related; combining principles of both can facilitate more efficient, equitable progress toward team outcomes, improved population health, and increased health equity. This model could assist researchers and public health practitioners in designing community-relevant, scientifically rigorous research with practical implications for improving health and quality of life among marginalized populations.

## Introduction

Public research investment has expanded into two new and promising research approaches to addressing health disparities; one of these is transdisciplinary (TD) research and the other is community-based participatory research (CBPR) [1–6]. TD research from a health perspective aims to incorporate and integrate concepts from multiple disciplinary perspectives with the scientific goal to develop new theories, methods, or frameworks that transcend any single discipline, and a population health goal of more effectively addressing and solving complex health-related societal problems [5,7]. TD research in public health generally reflects a problem-centric, rather than discipline-centric approach to achieve that goal. Some definitions of TD research specify that partners or practitioners from non-scientific backgrounds can be involved in its conduct [5,7]. CBPR is an ecological approach seeking to give voice to marginalized communities by promoting engagement with community members at each phase of the research process, from identification of the problem to be addressed to conceptualization of community-relevant research questions, study design, analysis, and interpretation. The approach aims to address problems of relevance to specific communities [8]. Both TD and CBPR focus on promoting equity, social justice, and elimination of health disparities [9,10]. Table 1 describes key characteristics of each approach [11–13].

**Table 1.** Key characteristics of transdisciplinary and community-based participatory research

**Transdisciplinary research [7,11,12]**
Performed with explicit intent to solve multi-dimensional, complex problems
Involves a changing methodology to respond to problem being investigated
Integrates knowledge and expertise from multiple academic disciplines, culminating in new theories, methods, and/or frameworks that transcend involved disciplines
May involve partners or practitioners from non-scientific backgrounds
**Community-based participatory research [13]**
Recognizes community as a unit of identity
Identifies and builds upon community strengths and resources
Facilitates collaborative, equitable involvement of all partners in all phases
Integrates knowledge and action for benefit of all partners
Fosters empowering co-learning among partners that addresses social inequality
Employs iterative approaches to support partnerships
Addresses problems defined by the community from positive and ecological perspectives
Disseminates knowledge widely
Requires partnersʼ long-term commitment

Peer-reviewed literature that describes the explicit integration of CBPR and TD research is scarce, and the research that exists has been largely theoretical. For example, Dankwa and colleagues proposed integration of translational, TD, and transformational research approaches with respect to addressing health disparities, but did not explicitly integrate CBPR into the model [14]. Recent work by Wallerstein and colleagues describes a single Clinical Translational Science Award project as an example of integrating team science and different levels of community engagement to address complex public health and clinical problems [15]. However, no prior work to our knowledge has explicitly examined the practice of integrating TD research and CBPR approaches across multiple centers and research projects.

The Centers for Population Health and Health Disparities (CPHHD), a collaborative, multi-institution initiative funded by the National Institutes of Health (NIH) between 2003 and 2015, attempted to integrate TD research and CBPR. It was designed to foster TD science among biological, medical, behavioral, social, and population health researchers and to incorporate principles of CBPR and community engagement to address health inequities, with respect to cancer and cardiovascular disease [16]. The explicit expectation that CPHHD teams integrate TD and CBPR in their approaches to research provided an opportunity to examine how a combined TD and CBPR strategy was conceptualized and implemented. In this study, we used a multi-method case study approach to assess how CPHHD investigators formed the integrated TD and CBPR approach in their work, and what they perceived as the benefits of that work and its impact on health disparities. This empirical evidence could assist researchers and public health practitioners in designing community-relevant, scientifically rigorous research with practical implications for improving health and quality of life among marginalized populations.

## Methods

### Setting

The CPHHD, a P50 specialized center grant, serves as a case focus here. Specialized center grants fund research and development projects across multiple institutions to utilize multidisciplinary team-based approaches for addressing a specific public health need identified by an NIH institute or division. Supported by the National Cancer Institute (NCI), the National Heart, Lung, and Blood Institute (NHLBI), and the Office of Behavioral and Social Sciences Research, the CPHHD fostered multi-level, community-engaged, TD research that addressed disparities in cancer and cardiovascular disease risk and outcomes [2,6]. The initiative required investigators to incorporate *both* principles of CBPR and TD research and to conduct at least one multi-level, community-engaged intervention. As a CPHHD requirement, study teams comprised investigators from universities and comprehensive cancer centers as well as individuals who represented various academic disciplines and diverse community, health care practice, and policy groups who collaborated to integrate TD and CBPR approaches in their work in service of addressing health disparities.

The first 5-year grant cycle of the CPHHD was established in 2003; the focus of the present work is on the CPHHD centers funded in the second 5-year cycle (2011–2015), whose projects are listed in Table 2. Investigators at 10 research institutions focused on integrating scientific, community-focused approaches to better understand and reduce health disparities. The program prioritized the establishment of a comprehensive TD framework that emphasized a common research thread from basic, clinical, and population science. A key aspect of the funded projects was to develop interventions by partnering with members from diverse communities and to identify practices and policies that reduce health disparities [2,17,18].

**Table 2.** Centers for population health and health disparities research centers and project titles, 2011–2015

Institution	Project title
*National cancer institute-funded centers*	
Fred Hutchison Cancer Research Center, Seattle, Washington	Understanding and Preventing Breast Cancer Disparities in Latinas
Harvard University School of Public Health, Boston, Massachusetts	Lung Cancer Disparities Center: Jointly Addressing Race and Socioeconomic Status
Johns Hopkins University, Baltimore, Maryland	Hopkins Center for Eliminating Cardiovascular Health Disparities
Northeastern University, Boston, Massachusetts	Boston Puerto Rican Health Study - CVD Risk Factors
Ohio State University, Columbus, Ohio	Reducing Cervical Cancer in Appalachia
*National heart, blood, and lung institute-funded centers*	
Rush University Medical Center, Chicago, Illinois	Rush Center for Urban Health Equity Diet and CVD in Puerto Ricans
University of California, Los Angeles, California	Family and Neighborhood Interventions to Reduce Heart Disease Risk in East L.A.
University of Illinois at Chicago, Chicago, Illinois	UIC Center for Population Health and Health Disparities, Breast Cancer Diagnosis and Treatment
University of North Carolina, Chapel Hill, North Carolina	Center for Reduced CVD Disparities: Genes, Clinics, and Communities
University of Washington, Seattle, Washington	Center for Native Population Health Disparities

CVD, cardiovascular disease; L.A., Los Angeles.

### Study Design

This work was guided by three overarching questions: 1) How did CPHHD centers integrate TD research and community-engaged research? 2) What did investigators perceive as the benefit of their TD, community-engaged work? and 3) What did they perceive as the impact of that work on the field of health disparities? We utilized a qualitative, convergent parallel case study design, in which different, complementary data are collected simultaneously; data integration occurs during analysis and interpretation [19]. To attain the convergent parallel case design, we conducted a document review of the NIH-issued CPHHD Request for Applications (RFA), each funded center’s research project abstracts, and scientific meeting agendas to better understand the stated requirements and contexts of the research projects [20]. To complement document data and to better understand complex, multi-dimensional issues, we conducted semi-structured interviews with CPHHD investigators and funding agency staff.

### Document Review

We downloaded RFAs from the NIH website [16] and obtained scientific meeting agendas and project abstracts from CPHHD leadership. Two members of the study team first reviewed each RFA and all meeting agendas. In consult with the senior author, we created an Excel document to summarize characteristics of the initiative, specific requirements for TD and community-engaged research, and descriptions of how investigators intended to conduct TD, community-engaged work based on project abstracts. In accordance with the parallel convergent study design, we integrated analyses of the document review findings, interpretation, and presentation alongside interview findings in the results.

### Interviews

A nine-question interview instrument was developed and pre-tested among investigators from a different TD center-grant initiative and approved by CPHHD Steering Committee members. Questions aimed to ascertain specifically how CPHHD perceived their projects to have incorporated TD and community engagement approaches into their research projects, the benefits of integrating TD and CBPR, and the impact of the integrated approach on health disparities. We applied purposeful, maximum variation sampling to recruit investigators who represented all 10 CPHHD centers and the primary funding agencies (NCI and NHLBI). Maximum variation is an approach in which a small number of interviews are conducted to maximize divergent experiences and perceptions relevant to the research question [21]. Maximum variation enabled us to identify common patterns and diverse variations of processes and outcomes across CPHHD interview participants [21,22]. We contacted potential respondents via a recruitment email explaining the study, and follow-up phone calls when needed, to schedule one-on-one interviews. Respondents provided oral consent to audio-recorded telephone interviews and received a $25 gift card. Two study team members were present during interviews: one to conduct the interview and another to record notes. Interviews were transcribed, checked for accuracy, de-identified, and uploaded into Atlas.ti (Version 8) for coding. The Fred Hutchinson Cancer Research Center IRB and the CPHHD Steering Committee approved the study.

### Interview Analysis

**Fig. 1.**
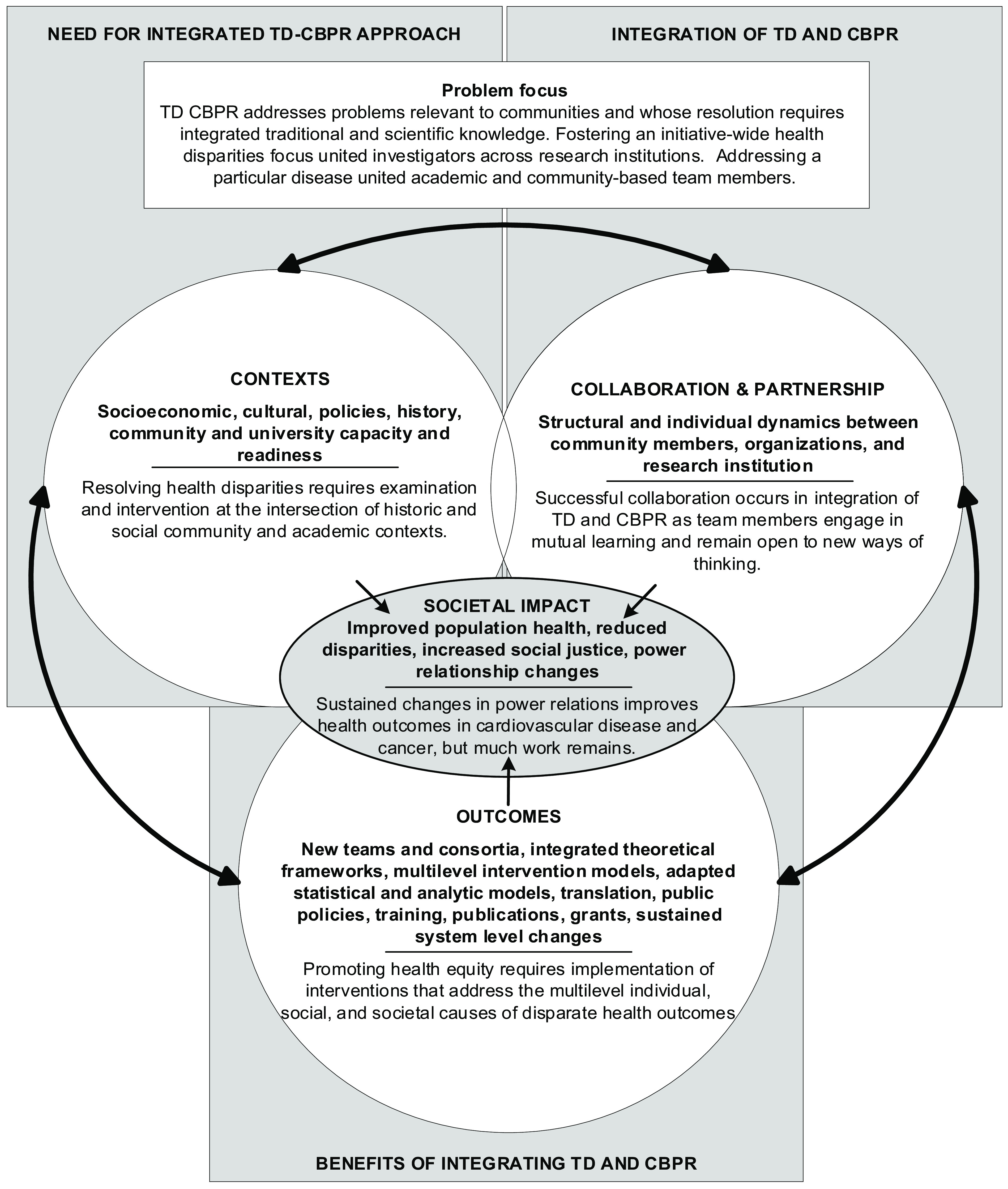
Working conceptual model of transdisciplinary (TD), community-based participatory research (CBPR). Bold text indicates codes applied to qualitative interview data, which are examples of each construct adapted from Wallerstein and Duran (2016), Warnecke (2008), and Hohl (2020). Non-bold text provides a summary of each construct based on the current analysis.

Our analysis was guided by the three research questions described above. Codes were developed both *a priori* based on Kastelic and colleagues’ CBPR conceptual model [23]. Warnecke and colleagues’ model for analysis of population health and health disparities [6] and our team’s previous work investigated TD outcomes [24], and deductively, based on interview and document data. These codes are reflected in our working conceptual model (Fig. 1). We applied a constant comparison analytic approach, the goal of which is to generate theory by iteratively comparing emergent concepts from each data source to those coded and analyzed previously and subsequently [25]. We reviewed coded text and selected representative quotes from interviews that best summarized each code.

## Results

We reviewed 1 RFA, 5 scientific meeting agendas, and project abstracts for all 10 CPHHD centers. Between July and August 2015, 25 CPHHD investigators and NIH program staff participated in a telephone (n = 23) or face-to-face (n = 2) interview lasting between 37 and 50 minutes. Respondent characteristics are described in Table 3. Our original research questions focused on exploring integration, benefits, and impact of combining TD and CBPR, but analysis revealed these topics could not be adequately explored or described without also examining the need for and context in which these approaches were combined. Therefore, the results are organized into four categories: 1) the necessity of TD and CBPR; 2) tntegrating the approaches; 3) benefits and outcomes; and 4) impact. We provide illustrative quotes embedded within a summary of those categories.

**Table 3.** CPHHD interview respondents. July–August 2015

Respondent role	n
Center Director or Co-Director	13
Project investigator or core leader	5
Early career investigator	4
Funding agency representative	3
Total	25

CPHHD, Centers for Population Health and Health Disparities.

### The Need for TD CBPR Approaches

#### Problem focus

The CPHHD was created with a goal to address health disparities in a range of marginalized communities throughout the USA. As summarized by this participant, *“Health disparities really goes beyond a particular disease. It goes to social justice, and the impact of social justice on people’s health.”* **(Participant 2)** Accordingly, all project abstracts demonstrated that centers in the initiative focused on disparities in either cancer or cardiovascular disease across a broad range of racial, ethnic, socioeconomic, and geographic groups. All but four respondents – who spanned CPHHD centers and funding agencies – suggested that while a unifying focus on one disease area facilitated a common goal among the community groups with which they partnered, it also highlighted differences among investigators across the initiative itself. This participant’s sentiment that the common thread across projects was not diseases, but patterns of disparities, was shared widely across the CPHHD:

*“Since this is a partnership between cardiovascular health and cancer, it’s impossible to put things together, firstly because their outcomes are different. […] And the interventions proposed [across centers] are different. […] You cannot do a one-on-one comparison. You can only talk about patterns by which disparities and outcomes occur.*” **(Participant 13)**

#### Contexts

Throughout interviews, all respondents reflected on the historical and social contexts that have and continue to influence health disparities. They said understanding those contexts are integral to developing interventions and framing solutions to the public health challenges they set out to address. This investigator summarized:

*“When you think about what impacts health disparities, you quickly get to the whole idea of multilevel causation. There’s the environment, biology, education and income, access to resources, and you know, what’s around you, who youʼre friends with, and what your family members do, in terms of health behaviors. […] It was our framework that we canʼt intervene at one level and expect to have a big impact.”* **(Participant 18)**

TD research processes are influenced heavily by contextual factors such as institutional resources and organizational structure, including university or cancer center support for team science and collaborative investigation. The CPHHD investigators described the intersection of the historical and social contexts that drive disparities *and* the academic contexts that influence if and how community-engaged, TD research is implemented. Seventeen (about two thirds) of respondents widely discussed the lag in academic institutional policies to support addressing contemporary public health problems as a primary barrier to implementing TD, community-engaged research:

*“The scientists who were working within these transdisciplinary enterprises—the [Transdisciplinary Tobacco Use Research Centers], the [Transdisciplinary Research on Energetics and Cancer], the CPHHDs—have agreed or accepted and even appreciated this perspective. But the institution’s criteria such as promotion, tenure, credit for work is all still from the 20th century approach to science. I donʼt know that we can solve this in one generation. […] Yet, there is a slow shift. The very fact that I can have a geneticist come and talk about racism in a project meeting with us says a lot about the acceptance.”* **(Participant 25)**

Moreover, these 17 investigators said their work in this type of initiative challenged the silo-orientation of traditional academic systems to develop sustainable infrastructure to address health disparities. This investigator explained:

*“Weʼre trying to get university funding for this new center. It’s going to focus on transdisciplinary initiatives and disparities […] We’ve brought in genetics and clinical medicine, epidemiologists, and pathology. Our outreach programs—which most of them are because you canʼt do this kind of research without having community partners—grew out of CPHHD funding and eventually merged into this Consortium, the Task Force that we now have. For more than ten years, weʼve been building on this, working with community partners to build a relationship that we could carry into this program.”* ***(*Participant 2)**

### TD CBPR Integration

#### Collaboration and partnerships

The requirement that projects utilize principles of CBPR necessitated investigators to develop and work in partnership with a wide range of community representatives in all phases of the research. The TD nature of the initiative also required collaboration across diverse academic disciplinary perspectives, resulting in complex systems that investigators said presented time-consuming, yet generally surmountable, difficulties, such as implementing protocols for collaboration and co-authorship on publications, establishing a common language, and building trust. Over three quarters of respondents representing CPHHD centers discussed the outcomes of their partnership development (e.g., task forces, community advisory boards, working groups) that, in most cases, took months and – more often – years to develop. These investigators explained:

*“We did a year of formative work and planning [with the community members] and with patients, to try to understand more about their perspectives. That allowed us to really tailor [our study] to the needs of both the intervention delivery system and the patients*.” **(Participant 6)**

*“I went out and did a ton of interviews, talking with providers, informatics folks, nurses, patients, people in the hospital, people in the emergency room, people at McDonald’s, to really get a sense of what the need was in the community.”* **(Participant 14)**

Respondents noted that despite the added time and effort, the integration of CBPR and TD approaches propelled them to engage in mutual learning with community partners and researchers and to think differently about the problems they were addressing. They described the critical importance of honoring different “ways of knowing” and different knowledge orientations. For example, this investigator commented on shortcomings in stress measurement for an urban Puerto Rican population,

*“We started thinking differently about the kind of stress that the urban poor experience than just the Perceived Stress Scale and things that are probably more appropriate for more affluent white populations.”* **(Participant 7)**

Reflecting the positionality of an academic researcher and the impacts of this type of research collaboration, this investigator summarized the benefit of mutual learning that resulted from developing partnerships beyond a single discipline and beyond academic institutions:

*“I’ve learned so much about genetics and its importance, and gene-nutrient interactions, and population stratification, things that I might not have had I not been working in this transdisciplinary environment. Our team [includes] someone who’s an expert both in air pollution and in CBPR. His perspective and insistence on involving the community and his knowledge of how to enter communities and things has been so valuable in our studies. […] This type of collaboration requires new approaches and new thinking, and it’s been this continual mutual learning process, a fantastic growth opportunity for the whole team.”* **(Participant 9)**

Similarly, an investigator who identified as a member of the community served by the research projects noted:

*“I’m speaking as a tribal person myself. We’re exploring what an all-Lakota project team can look like, and how the differing epistemology that is at play in traditional society here among the Lakota, for instance, compares with or is different from a more science-based epistemology.”* **(Participant 12)**

All investigators reflected that honoring the CBPR principle of building on strengths and resources of the community was critical to developing meaningful interventions with potential for sustainable reductions in health disparities. In addition to developing advisory boards inclusive of academic researchers, providers, and community members, CPHHD projects aimed to build local capacity, for example, by engaging and training community members to participate in intervention design and implementation. This investigator reported:

*“[We need to] select people with a track record of honoring the strengths of the communities they claim to serve […] Wherever you go, communities have tremendous assets.[…] Part of the reason why I think it’s very important that we rely more heavily on community health workers, rather than clinically-trained change agents when it comes to behavior change, is because their knowledge of the community allows them to be resource navigators. They can point out what the assets are to other members of the community, in a way that a physician or an RD [Registered Dietitian] trained at the university might not.”* ***(Participant 15)***

### Benefits of a TD CBPR Approach

#### Improved scientific and collaborative outcomes

Investigators described both TD research and CBPR outcomes such as integrated theoretical frameworks, changes in power relations, inclusive and multi-level intervention models, adapted analytic approaches, and sustained practices and policies to promote health equity. Of these outcomes, all CPHHD respondents consistently highlighted the benefit of the multi-level interventions that resulted from their TD CBPR approaches. Specifically, they suggested that by valuing and integrating different knowledge traditions from both within and beyond their research institutions, they were positioned to develop interventions with potential for meaningfully promoting health equity. This respondent summarized:

*“[The CPHHD] proves that multilevel intervention is possible to do. It shows that we need to intervene …not only at the individual level, but we need to look at the structural factors. […] and that the partnerships are so important. Most of the success of these programs are because of those partnerships with communities.”* **(Participant 13)**

Investigators emphasized the role of partnerships and infrastructure supported by the CPHHD that sparked new ideas and further sustained relationships with community partners. This investigator described how integrating academic and biblical orientations culminated in a new grant proposal to address a community health concern, promoting agency among the pastors who advocated for the intervention:

*“The infrastructure we had from the CPHHD made it possible for us to respond to a request that we got from the black ministers […who] said, ‘We’re really worried about the stress in our lives and its implications for our own personal health. Can you help us?’ […] As we were working with them intensively, […] they were starting to bring in insights about health from the Bible. We never thought that there would be this close correspondence between health promotion efforts and readings from the Bible, but they started talking about the intersection between spiritual development and health promotion, and that gave rise to the grant that we then got funded.[…] It didnʼt come from us; it came from the pastors.”* **(Participant 7)**

For about half of investigators, adhering to the CBPR principle of sharing findings and knowledge gained with all partners, combined with the TD approach of involving multiple academic and community stakeholders, represented a shift toward more relevant, impactful research dissemination. This investigator reported:

*“With all of the CBPR, we have a better sense of what the community really needs and wants to see. The importance of the contributions that will come from this have been greatly enhanced by this transdisciplinary approach. […] There are more people who see value in it than it would have if it was just some clinicians hanging around trying to do another study. It’s more valuable to the community for sure. […] We went back out into the community to make sure they saw the data from the three studies. […] We did newsletters that went to all the patients involved in the practices, we brought these real-life stories of people who really got their blood pressure down […] to highlight the participants. We’d put one of [name]’s great heart healthy recipes in there, so much more digestible products back to the community than what we would have done in a standard RCT done ten years ago.” (* ***Participant 14)***

### Impact of TD CBPR on Health Equity

#### Societal impact

The CPHHD’s stated desired societal impact was improved cardiovascular disease and cancer outcomes. Eleven investigators (almost half) related that despite the unified expertise of investigators, community members, and health care providers, some interventions did not have the intended effect, demonstrating the complicated, deeply embedded, and profound impact of disparities on health outcomes. These investigators considered, if their CBPR approach did not work, what next to address health disparities? This investigator questioned:

*“I think what happens a lot is in CBPR you get everybody involved, all of the partners, the ideology of how you put the team together and the democratic approach to every step of the way, I think that’s really exciting. But if, with all of that background work, we still donʼt get the results that we were hoping to get, what do we do as the next step? I think that’s a really important question in this field.”* **(Participant 7)**

Despite these sentiments, the 16 investigators from institutions that had participated in both cycles of CPHHD funding, as well as those with pre-existing institutional support for collaboration and CBPR approaches described multiple advances in bolstering social justice and health equity, and reducing disparities that resulted from their TD approaches. These investigators described:

*“CPHHD projects are a new kind of science that represents this transdisciplinary approach. Most of the things that have been done by the Centers have been done in a multidisciplinary capacity. The CPHHD has focused attention on multiple levels, which is a difficult thing to implement in practice, but weʼre moving closer to being able to do that. […] our case, weʼve actually had an impact on breast cancer mortality in Chicago.”* ***(Participant 2)***

*“Our goal was to reduce cervical cancer in Appalachian women. By doing all this transdisciplinary work in the community, we’ve been able to learn [that] not only are cervical cancer rates elevated in Appalachian women, the high-risk HPV rates are elevated. There is an increased risk because of [gene] mutations. We know smoking rates historically are high in Appalachia and HPV vaccination rates are low […] All of that has an effect and so [we] intervened to increase the vaccination rate.”* ***(Participant 3)***

## Discussion

In this study, we described CPHHD members’ efforts to integrate TD and CBPR, the benefits of such an integration, and its impact on health equity. Figure 1 represents a visual integration of the concepts that emerged from our analysis and adaptations of the CBPR conceptual model [23] and that incorporated the complementarity of the two approaches. We posited that social, cultural, and historical contexts of research institutions and community groups; structural and individual dynamics between communities and research groups; and desire to achieve public health and research outcomes would influence each other to generate societal impact (i.e., improved population health, reduced disparities, and increased social justice and health equity). The model is iterative in nature and is thus depicted in a circular rather than linear form, wherein the problem focus of the research – which reflects a priority health issue identified by community members – influences contexts, collaboration and partnership, and outcomes of this type of work. These factors in turn influence the societal impact (e.g., reduced health disparities) that can emerge from a TD CBPR approach to health disparities. In our model, we categorized concepts such as sustained changes in power relations and cultural renewal, reduced health disparities, and increased social justice as impacts to society.

Investigators additionally described the complex social and environmental contexts such as university and cancer center policies, availability of collaborative infrastructure and the history of marginalization among communities that influenced their TD, community-engaged health disparities research projects. Several individuals highlighted the immense time and effort required to conduct truly collaborative, community-based research, but saw its rewards as valuable – and critical – to both academic institutions and the well-being of all populations. Investigators described the essential role of spending time building lasting relationships with community partners to better understand communities’ health needs, jointly develop interventions, and disseminate meaningful information during and upon study completion. They highlighted the mutual learning, a key construct of both transdisciplinarity [26–28] and CBPR [29–31], that occurred across academic and community disciplines and positioned teams to address multiple levels of influence on health.

Previous CBPR work has emphasized the importance of decolonizing and democratizing knowledge, that is, valuing epistemology (i.e., local ways of knowing) and ontology (i.e., community truth and understanding) [32–35]. These concepts are practiced by seeking to understand indigenous theories and by recognizing their value in solving community-relevant public health problems [32,35], by conceding power and decision-making that has traditionally been held exclusively by academic researchers to community members [33], and by challenging academic institutional norms, such as Institutional Review Board processes that may not align with CBPR principles of community engagement in research [34]. The attitudes of many investigators interviewed reflected an orientation toward epistemic justice, wherein credibility of community members’ knowledge and intelligence was acknowledged and championed [36] as they contributed to all phases of research projects. System-level change, such as a shift in power relations and cultural renewal, is an identified outcome of CBPR [31]. Investigators in previous work have identified university context and support in general as integral to the success of collaborative TD research [37–39].

### Recommendations for Adopting the TD CPBR Approach

Table 4 outlines recommendations for research teams looking to adopt the TD CBPR approach, organized by level of the Working Conceptual Model illustrated in Fig. 1. Our recommendations offer suggestions for navigating the process of planning a project using the TD CBPR approach, beginning with expecting the significant time investments that TD CBPR requires. In addition to practical strategies to implement the approach (e.g., define the role of power relations in achieving the societal impact the team aims to realize; identify concrete strategies team members will employ to shift power dynamics), the recommendations emphasize measurement and evaluation of that process (e.g., measure progress as the team implements those strategies to shift power dynamics). Although these recommendations are not exhaustive, they provide a foundation from which teams may begin to build their TD CBPR projects.

**Table 4.** Recommendations for teams adopting the TD CBPR approach, organized by level of the working conceptual model of transdisciplinary, community-based participatory research

**Societal impact**
Expect that both achieving societal impact through the TD CBPR approach and the process of doing so requires significant time investment
Define the role of power relations in achieving the societal impact the team aims to realize
Identify concrete strategies team members will employ to shift power dynamics
Measure progress as the team implements those strategies
**Contexts**
Identify the multi-level individual, social, and societal causes of health outcomes
Become familiar with the historic and social community and academic contexts that have contributed to imbalances in power, privilege, access to resources, and health inequity
Consider the implications of those contexts on the process and outcomes of the projects undertaken
Identify strategies team members will use to address these historic and social contexts
Measure progress as the team implements those strategies
**Collaboration and partnership**
Remain open to new ways of thinking
Consider how those ways of thinking can be integrated to create new, inclusive, and holistic approaches to community-relevant research
Explicate how decision-making will be shared across collaborators
Identify strategies team members will use to incorporate alternative ways of thinking into their projects
Measure progress as the team implements those strategies
**Outcomes**
Measure progress toward integration and development of new, inclusive approaches to research

There are several limitations of this work. First, the working conceptual model relies on the voices of investigators within the CPHHD and data sources, such as project abstracts and initiative meeting agendas that were designed for investigators themselves. Importantly, only two community partners’ perspectives are reflected in this work. However, their responses aligned with those of academic investigators. A more in-depth examination of community members’ perspectives of partnership, empowerment, equitable involvement, and disciplinary/epistemic integration could build on this work. Future studies should engage community members in refining this model, as was done for Wallerstein’s model of CBPR [29]. Further, part of the analysis relied on what was described in project abstracts from grant proposals, which may not have reflected the research ultimately conducted, given the need for CBPR researchers to respond to the needs of communities.

Despite these limitations, this work benefitted from several strengths. First, we utilized multiple data sources that included the RFA, project abstract, meeting agendas, and in-depth qualitative interviews across 10 research centers and 2 NIH funding agencies. The range of investigator perspectives across a nationwide publicly funded initiative provided a broad understanding of the practical application of the combined TD and CBPR approach to reduce health disparities in cancer and cardiovascular disease. Interview respondents represented diverse disciplines and partnered with communities that included various racial, ethnic, socioeconomic, and geographic subgroups that experienced myriad health challenges related to cancer and cardiovascular disease.

## Conclusion

The recognition by Western funding agencies that community participation in research and social action is essential to ameliorating complex health disparities is reflected in increased funding for and prioritization of CBPR and community engagement in research and practice [23,40–42]. Scholars involved in many of these community projects have developed conceptual frameworks to guide planning and evaluation of TD research [27,39] and CBPR [23,29,31]. To date, models of TD research processes and outcomes for public health problems have focused almost exclusively on the knowledge, evidence, and outcomes generated through the integration of traditionally recognized academic disciplines [27,39], which is important for scientific advancement but may not always be relevant for the communities that such work aims to benefit. Models of CBPR have focused on the processes and outcomes of engaging community stakeholders, democratizing knowledge, and tailoring research projects to specific and relevant contexts [23,29,31]. As stakeholders in research and community organizations recognize the value of and begin to fund and implement integrated TD and community-engaged research, this work represents a step toward a holistic understanding of their intersection in practice. This work could help guide planning, implementation, and evaluation of TD CBPR projects and ultimately enhance potential to improve health among marginalized communities.
